# The utility of 4D intracardiac echocardiography in transcatheter pulmonary valve replacement in adult congenital heart disease patients

**DOI:** 10.1016/j.ijcchd.2026.100652

**Published:** 2026-01-06

**Authors:** Eihab Ghantous, Hans Gao, Carlos Sisniega, Angela Li, Jamil Aboulhosn

**Affiliations:** a Ahmanson/UCLA Adult Congenital Heart Disease Center, Division of Cardiology, David Geffen School of Medicine at UCLA, USA; b Division of Pediatric Cardiology, David Geffen School of Medicine at UCLA, USA

**Keywords:** Transcatheter pulmonary valve replacement, Intracardiac echo, Tetralogy of fallot, Pulmonary regurgitation

## Abstract

**Background:**

Intracardiac echocardiography (ICE) has become a critical imaging modality in adult congenital heart disease (ACHD) interventions, offering high-resolution intracardiac imaging without the need for intubation. In transcatheter pulmonary valve replacement (TCPVR), ICE may overcome limitations of traditional imaging, particularly in visualizing the right ventricular outflow tract (RVOT) and pulmonary valve.

**Objectives:**

This study aimed to evaluate the feasibility, safety, and clinical utility of four-dimensional (4D) ICE in ACHD patients undergoing TCPVR.

**Methods:**

Beginning in February 2023, we prospectively enrolled all patients undergoing TCPVR at a tertiary ACHD center. Patients underwent preprocedural imaging and clinical evaluation. 4D ICE was used pre- and post-valve implantation to assess cardiac anatomy and valve function. Patients without valve implantation or 4D ICE imaging were excluded.

**Results:**

Of 55 referred patients, 45 underwent successful TCPVR with 4D ICE. The mean age was 42.6 ± 13.6 years, and 51 % were male. 4D ICE confirmed procedural indications, provided comprehensive anatomic and functional assessment, and detected post-implantation regurgitation in 35.6 % of cases, significantly more than angiography (11.1 %) or transthoracic echocardiography (4.4 %). Incidental but clinically relevant findings were identified in 33.3 % of patients, influencing management in 15.6 %. No ICE-related complications occurred.

**Conclusions:**

4D ICE is a feasible, safe, and clinically valuable imaging tool during TCPVR in ACHD patients. It enhances procedural guidance, detects early valve-related complications, and identifies unexpected findings that affect management. Its integration into structural heart interventions may significantly improve outcomes, especially as technology continues to evolve.

## Highlights

• 4D ICE is feasible and safe during TCPVR in ACHD patients.
• 4D ICE is more sensitive than angiography or TTE in detecting PR.
• ICE identified incidental findings in one-third of patients.
• Incidental findings altered management in 15.6 % of cases.
• 4D ICE provided enhanced visualization of pulmonary valve anatomy.

## Graphical abstract

**Figure:**
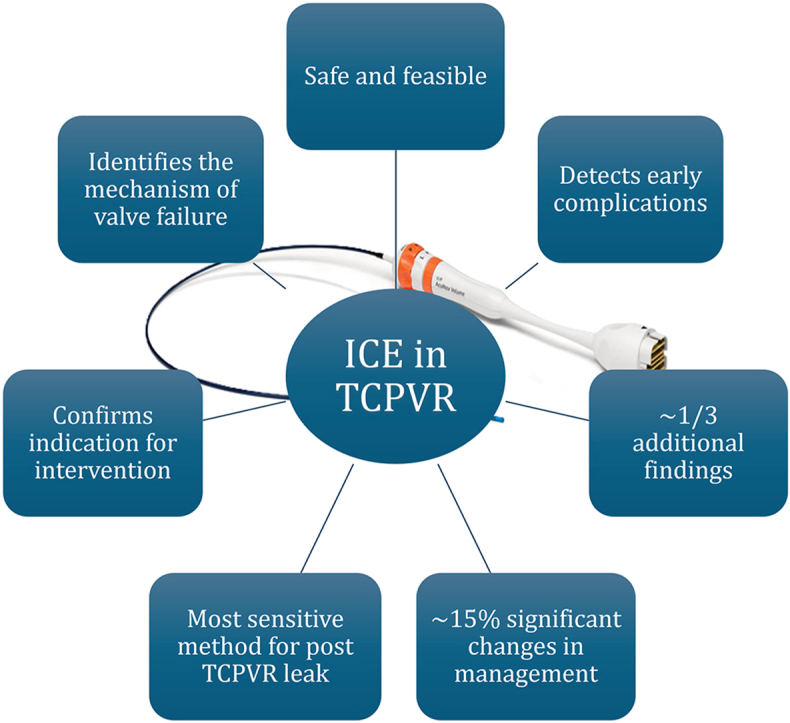

## Abbreviations:

ACHD –: Adult congenital heart disease
ICE –: Intracardiac echo
RVOT –: Right ventricular outflow tract
TCPVR: Transcatheter pulmonary valve implantation
TEE –: Transesophageal echocardiography
ToF: Tetralogy of Fallot

## 1 Background

Intracardiac echocardiography (ICE) was introduced in the 1960s [1] and has since evolved into an integral imaging modality for various percutaneous interventional procedures in both the adult cardiology and the adult congenital heart disease (ACHD) population [2]. Its growing use is attributed to several advantages, including the ability to obtain high-quality intracardiac imaging without the need for endotracheal intubation or endoscopic intubation, elimination of the risk of esophageal trauma, reduced sedative requirements, and the ability to perform procedures without requiring an additional operator [3,4].

Among patients with ACHD, a significant proportion have undergone surgical interventions involving the right ventricular outflow tract (RVOT), often utilizing conduits and prosthetic valves [5,6]. Over time, these conduits and valves are prone to dysfunction, necessitating repeat interventions, either surgical or percutaneous. In those that underwent surgical treatment of RVOT disease without placement of a conduit or percutaneous valve, most are left with significant pulmonary valve regurgitation that eventually necessitates pulmonary valve replacement. Transcatheter pulmonary valve replacement (TCPVR) was first introduced in 2000 [7], and has since gained widespread acceptance, becoming the most frequently performed transcatheter valve procedure in patients with ACHD [[8], [9], [10], [11]]. Historically, these procedures relied heavily on fluoroscopy and angiography for procedural guidance due to the limitations of transthoracic echocardiography (TTE) and transesophageal echocardiography (TEE) in visualizing the RVOT conduit and pulmonary valve. However, recent advancements in ICE technology have opened new avenues for its application in TCPVR, offering superior visualization of intracardiac structures and procedural guidance [2,9,[12], [13], [14]].

Herein, we present our experience with the use of four-dimensional (4D) ICE in ACHD patients undergoing TCPVR, highlighting its feasibility, added clinical value, and potential future applications.

## 2 Methods

Patient population: Beginning in February 2023, we prospectively enrolled all patients undergoing TCPVR at the Ahmanson/UCLA Adult Congenital Heart Disease Center. All procedures were performed by a single primary operator. Each patient underwent comprehensive preprocedural planning, which included advanced imaging with cardiac computed tomography (CCT) or cardiac magnetic resonance imaging (CMR), TTE, and an outpatient clinical visit for a thorough discussion of available valve options prior to catheterization. Patients were excluded if the procedure did not include valve implantation or if 4D ICE imaging was not performed.

### 2.1 Transcatheter pulmonary valve replacement (TCPVR)

All TCPVR procedures began with a comprehensive right heart catheterization, including hemodynamic and saturation measurements. This was followed by a pulmonary angiogram using biplane fluoroscopy, and subsequent baseline ICE imaging (as detailed below). The valve implantation was then performed using either the Medtronic Harmony valve system or the Edwards Sapien valve in conjunction with the Alterra prestent, when indicated, as previously described [15,16].

### 2.2 Intracardiac echocardiography (ICE)

**Fig. 1:**
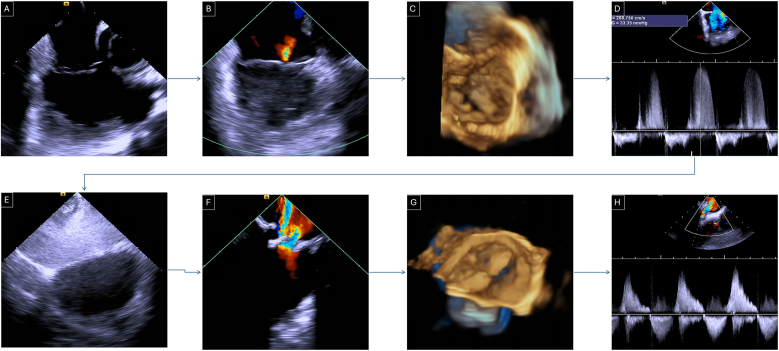
ICE protocol: A- “home view” looking at the tricuspid valve, RV function and pericardial effusion in 2D. B- Color Doppler on the tricuspid valve to check for TR. C- 3D image of the tricuspid valve. D- CW Doppler to check for tricuspid valve peak instantaneous gradient. E− Looking at the interatrial septum with agitated saline test showing agitated saline in the RA and clear LA. F- Looking at the pulmonary valve with color Doppler showing severe regurgitation. G- 3D of the pulmonary valve. H – CW across the RVOT. CW, continuous wave Doppler; ICE, intracardiac echocardiography; LA, left atrium; RA, right atrium; RV, right ventricle; RVOT, right ventricular outflow tract.

ICE imaging was performed using the Siemens AcuNav 4D ICE catheter, introduced through a minimum 14 Fr sheath placed in the femoral vein. Our imaging protocol (Fig. 1) began in the right atrium (RA), using the “home view” to visualize the tricuspid valve (TV), right ventricle (RV), and pericardium. We performed a qualitative assessment of RV systolic function, evaluated for pericardial effusion, and assessed the TV with and without color Doppler. Continuous wave (CW) Doppler was used to measure the peak instantaneous systolic TV gradient. We then rotated posteriorly to assess the interatrial septum in both 2D and 3D views, with and without color Doppler, followed by an agitated saline injection via the IVC to evaluate for right-to-left shunting. Manual compression of the abdomen was used to simulate a Valsalva maneuver during agitated saline injection. The ICE catheter was then advanced into the RV and directed toward the RVOT to assess the pulmonary valve (PV) using 2D and 3D imaging, with and without color Doppler, and CW Doppler for gradient measurements. Following valve deployment, we used the valve delivery sheath (26 Fr 65 cm long Dryseal sheath) positioned in the RVOT to facilitate advancement of the ICE catheter and performed post-implantation imaging in reverse sequence - starting in the RVOT to evaluate the newly implanted valve and moving back to the RA to reassess the tricuspid valve and pericardium. It should be noted that in the majority of cases additional fluoroscopy is not needed for placement or manipulation of the ICE catheter, 2D echo imaging is utilized for navigation throughout the procedure. Although not included in our TCPVR-specific ICE protocol, it is important to note that ICE allows comprehensive evaluation of additional intracardiac structures. From the home view, clockwise rotation of the catheter provides visualization of the mitral valve, portions of the left ventricle, the interatrial septum, and the left atrial appendage. When the catheter is advanced into the right ventricle, clockwise rotation enables imaging of the interventricular septum, left ventricular outflow tract, aortic valve, RVOT, and pulmonary valve.

### 2.3 Statistical analysis

Continuous variables with normal distribution were expressed as means ± standard deviation (SD). Normality was assessed using the Shapiro–Wilk test and visual inspection of quantile–quantile plots. Categorical variables were expressed with numbers with percentile and compared using the chi-square (χ^2^) test or Fisher's exact test, as appropriate. A p-value of <0.05 was considered statistically significant. All analyses were performed using IBM SPSS Statistics, version 26 (IBM Corp., Armonk, NY).

All authors contributed to the study design, data collection, statistical analysis, drafting, and revision of the manuscript. The study was approved by the institutional ethics committee (IRB# 11-001548).

## 3 Results

**Fig. 2:**
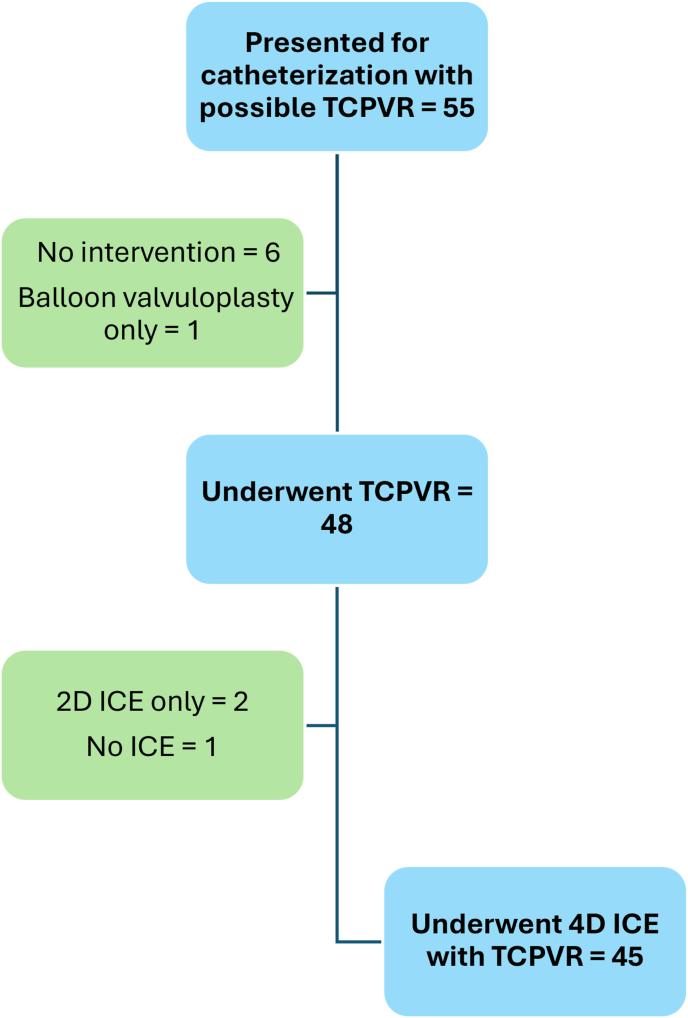
Patients included in the study.

Between February 16, 2023, and December 30, 2024, a total of 55 patients were referred to the catheterization laboratory at the Ahmanson/UCLA Adult Congenital Heart Disease Center with the intent to undergo TCPVR. Of these, 7 patients did not proceed with valve implantation, 2 patients underwent only 2D intracardiac echocardiography (ICE), and 1 patient could not undergo ICE imaging due to technical issues (Fig. 2). Ultimately, 45 patients underwent successful TCPVR with 4D ICE imaging pre and post valve implantation.

Patients’ characteristics are summarized in Table 1. The mean age at the time of the procedure was 42.6 ± 13.6 years; 51 % of patients were male. The most common underlying congenital cardiac diagnosis was tetralogy of Fallot (64.4 %), followed by valvular pulmonary stenosis (22.2 %). The indications for TCPVR were isolated pulmonary regurgitation in 51.1 % of patients, RVOT obstruction in 15.6 % (5/7 (71.4 %) had bioprosthetic valve and 2/7 (28.6 %) had pulmonary homograft), and mixed disease in 33.3 %. No complications related to the procedure or the use of 4D ICE occurred. This is confirmed from the pre- and post TCPVR ICE imaging findings detailed in Table 2.

**Table 1:** Patients' characteristics.

Variable	
**Age (years), mean ±SD**	42.6 ± 13.6
**Sex**	
• Male, n(%)	23 (51)
• Female, n(%)	22 (49)
**BMI (kg/m2), mean ±SD**	27.7 ± 6.7
**CKD, n(%)**	5 (11.1)
**Congenital diagnosis**	
• ToF, n(%)	29 (64.4)
• Valvular pulmonary stenosis, n(%)	10 (22.2)
• DORV, n(%)	3 (6.7)
• Post Ross procedure, n(%)	3 (6.7)
**Indication for intervention**	
• RVOT obstruction, n(%)	7 (15.6)
• Pulmonary regurgitation, n(%)	23 (51.1)
• Mixed disease, n(%)	15 (33.3)
**Landing zone type**	
• Native/Patched RVOT, n(%)	20 (44.4)
• Bioprosthetic valve n(%)	15 (33.3)
• Conduit/Homograft valve, n(%)	10 (22.2)
**Implanted valve type**	
• Sapien S3, n(%)	25 (55.6)
• Alterra/Sapien S3, n(%)	5 (11.1)
• Harmony TPV25, n(%)	15 (33.3)

BMI, body mass index; CKD, chronic kidney disease; DORV, double outlet right ventricle; ToF, tetralogy of Fallot.

**Table 2:** Pre and post catheterization imaging findings.

Variable	Pre-TCPVR	Post-TCPVR
**Pericardial effusion, n(%)**	0 (0 %)	0 (0 %)
**RV systolic function**		
• Normal, n(%)	37 (82.2)	40 (88.9)
• Mildly reduced, n(%)	8 (17.8)	5 (11.1)
**TR severity**		
• None, n(%)	1 (2.2)	0 (0)
• Trivial/Mild, n(%)	22 (48.9)	29 (65.9)
• Mild-moderate, n(%)	8 (17.8)	6 (13.6)
• Moderate, n(%)	10 (22.2)	7 (15.9)
• Moderate-severe, n(%)	3 (6.7)	2 (4.5)
• Severe, n(%)	1 (2.2)	0 (0)
**TR peak instantaneous gradient (mmHg), mean ±SD**	30.3 ± 13.9	27.1 ± 12.9
**PR severity**		
• None, n(%)	1 (2.3)	29 (64.4)
• Trivial/Mild, n(%)	2 (4.7)	16 (35.6)
• Mild-moderate, n(%)	0 (0)	0 (0)
• Moderate, n(%)	7 (16.3)	0 (0)
• Moderate-severe, n(%)	4 (9.3)	0 (0)
• Severe, n(%)	29 (67.4)	0 (0)
**RVOT peak gradient (mmHg), mean ±SD**	19.9 ± 16	12.2 ± 7.8

PR, pulmonary regurgitation; RV, right ventricle; RVOT, right ventricular outflow tract; TR, tricuspid regurgitation.

4D ICE was feasible and confirmed the diagnosis for intervention in all patients. It was the most sensitive modality in diagnosing post TCPVR regurgitation, identifying some degree of intra or para-valve regurgitation in 16/45 patients (35.6 %; 95 % CI 21.6–49.5 %) compared to angiography in 5/45 (11.1 %; 95 % CI 1.9–20.3 %) and early post-procedure TTE 2/45 (4.4 %; 95 % CI 0.0–10.5 %). The absolute risk difference for regurgitation detection was 24.4 % (95 % CI 7.7–41.2 %) for ICE versus angiography and 31.1 % (95 % CI 15.9–46.3 %) for ICE versus TTE. Corresponding effect sizes showed significantly greater detection with ICE, with risk ratios of 3.20 (95 % CI 1.28–7.99) and 8.00 (95 % CI 1.95–32.79). Fisher's exact test confirmed statistical significance for both comparisons (p = 0.0115 for angiography, p = 0.00036 for TTE, see Table 3). All detected regurgitation was graded as mild. Importantly, 4D ICE allowed for precise localization of the regurgitation site in all cases (see an example in supplementary Video 1): 5 (31.2 %) were valvular and 11 (68.8 %) were paravalvular. In contrast, with 2D ICE alone, the exact location of regurgitation was difficult to determine with certainty in 5 cases (31 %), all of which were paravalvular as confirmed by 4D ICE. Additionally, 4D ICE enabled visualization of all three valve leaflets in 84.4 % of the implanted valves.

**Table 3:** Statistical comparison of regurgitation detection rate between 4D ICE, angiography and early TTE.

Modality	Detection Rate	95 % CI	Risk Ratio vs ICE (95 % CI)	p-value^a^
**4D ICE**	16/45 (35.6 %)	21.6–49.5 %	Reference	–
**Angiography**	5/45 (11.1 %)	1.9–20.3 %	3.20 (1.28–7.99)	0.0115
**Early TTE**	2/45 (4.4 %)	0.0–10.5 %	8.00 (1.95–32.79)	0.00036

CI, confidence interval; ICE, intracardiac echo; TTE, transthoracic echo.

a Fisher's exact test, two-sided.

The following are the Supplementary data related to this article:

Supplementary data related to this article can be found online at https://doi.org/10.1016/j.ijcchd.2026.100652.

Supplementary Video 1: 2D with Doppler and 4D ICE views of the RVOT of a patient post TCPVR. The paravalve regurgitation (red arrow) is visualized only in some angles in 2D and while it is well visualized on the 4D image (the lower right frame) with the exact location outside the valve frame (green arrowhead). 2D, two dimensions; 4D, four dimensions; ICE, intracardiac echo; RVOT, right ventricular outflow tract; TCPVR, transcatheter pulmonary valve regurgitation.Video 1

**Fig. 3:**
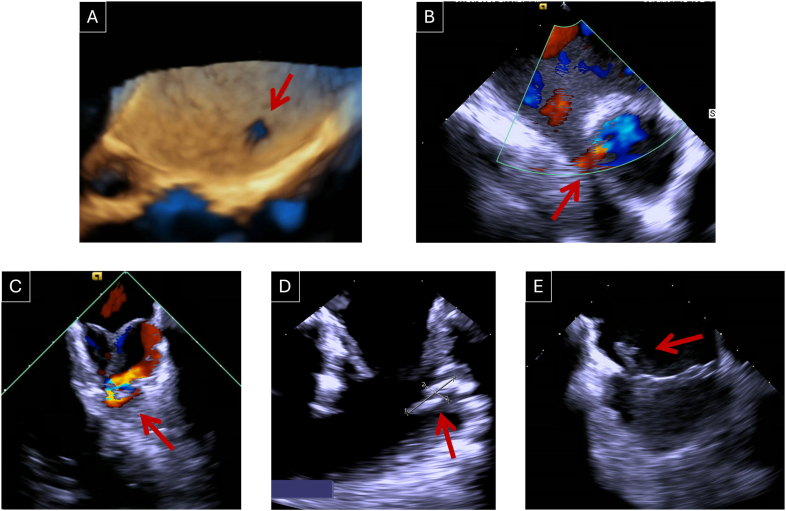
Additional findings indicated by red arrows: A- 3D view of an ostium secundum ASD. B- Color Doppler image showing unroofed coronary sinus with right to left shunt. C- Color Doppler image showing the aortopulmonary fistula. D- 2D image showing a thrombus in the MPA. E − 2D image of a thrombus on the pacemaker lead. ASD, atrial septal defect; MPA, main pulmonary artery.

Incidental and unexpected findings were identified by ICE in 15 patients (33.3 %), examples are shown in Fig. 3 and supplementary Videos 2-4. These included: 3 patent foramen ovales (PFOs), 5 ostium secundum atrial septal defects (ASDs), 1 unroofed coronary sinus, 1 aortopulmonary fistula, 1 thrombus on a pacemaker lead, and 4 thrombi in the main pulmonary artery. These findings influenced the management of 7 patients (15.6 %): 5 patients with thrombi were discharged on anticoagulation; one patient with an ostium secundum ASD underwent defect closure at the end of the procedure; and the patient with an unroofed coronary sinus was referred for cardiac surgery, with repair of the defect included in the surgical plan.

Supplementary data related to this article can be found online at https://doi.org/10.1016/j.ijcchd.2026.100652.

Supplementary Video 2: 2D with Doppler of the RVOT of a patient post TCPVR with an aortopulmonary fistula seen on color Doppler (red arrow). 2D, two dimensions; RVOT, right ventricular outflow tract; TCPVR, transcatheter pulmonary valve regurgitation.Video 2

Supplementary Video 3: 4D with Doppler ICE view of the interatrial septum showing an ostium secundum atrial septal defect (green arrow). 4D, four dimensions; ICE, intracardiac echo.Video 3

Supplementary Video 4: 2D ICE view of the RVOT of a patient post TCPVR showing an echodense structure at the RVOT compatible with a thrombus (red arrow). 2D, two dimensions; ICE, intracardiac echo; RVOT, right ventricular outflow tract; TCPVR, transcatheter pulmonary valve regurgitation.Video 4

The following are the Supplementary data related to this article:

## 4 Discussion

Since its introduction, ICE has significantly evolved and become an integral component of interventional procedures. Our study extends this evolution to TCPVR, demonstrating that 4D ICE is feasible, safe, and adds considerable value to the procedure.

At baseline, ICE enabled confirmation of the indication for intervention, detailed assessment of pulmonary valve function and the mechanism of dysfunction, evaluation of tricuspid valve function, and detection of pericardial effusion, thrombi, or vegetations on the pulmonary or tricuspid valves, as well as pacemaker leads. It also allowed for precise evaluation of the RVOT landing zone prior to valve implantation. Post-intervention, ICE proved to be an excellent tool for detecting early complications, including pericardial effusion, worsening tricuspid regurgitation, and assessment of the newly implanted pulmonary valve function and early thrombus formation.

4D ICE demonstrated superior sensitivity in detecting early valvular and paravalvular regurgitation with a three-to eight-fold higher detection rate compared with angiography and TTE accordingly. We believe that accurate localization and characterization of regurgitation, as enabled by 4D ICE, is clinically relevant and may inform future interventions, such as paravalvular leak closure if that is progressive.

Notably, the use of ICE resulted in the identification of incidental but clinically significant findings in one-third of patients. These findings directly impacted the clinical management of 46.7 % of those affected, influencing both post-procedural medication regimens and decisions regarding further interventions or follow-up strategies.

Although the use of 4D ICE requires at least a 14 Fr sheath, this does not increase the overall sheath size burden in TCPVR procedures, which routinely utilize sheaths larger than 22 Fr. Although we did not collect quantitative radiation data to compare cases with and without ICE, the procedures required minimal fluoroscopy because catheter navigation was performed under real-time 2D ICE guidance. Fluoroscopy was used only when necessary for localization.

Our findings build upon and expand previous work by Gonzalez de Alba et al., [13] who demonstrated the feasibility of 3D ICE in TCPVR. In contrast to their study, which utilized the Philips catheter and included pediatric patients with imaging performed only post-valve implantation, our registry included only adult patients (>18 years old) and utilized both pre- and post-implantation imaging with the Siemens catheter. This supports the generalizability of ICE across platforms and age groups.

While some findings in our study could be obtained using 2D ICE alone, we found that 4D ICE provided superior image quality, enhanced anatomical detail, and enabled a more comprehensive echocardiographic assessment, specifically of the newly implanted valve platform. Although the study was not designed to compare patient subgroups, 4D ICE appeared particularly advantageous in patients with paravalvular regurgitation or complex RVOT anatomy, where enhanced spatial resolution facilitated precise localization and characterization of the leak. Additionally, despite being bulkier, the 4D ICE catheter was not traumatic to surrounding tissues and safe to use. Although our study does not assess cost-effectiveness, we acknowledge that 4D ICE catheters are currently more expensive than 2D catheters; however, with advancing technology and broader adoption, costs are likely to decrease.

Our study has several limitations. It is a single-center experience conducted at a tertiary care facility, with all procedures performed by a single primary operator, which may introduce selection bias and may limit generalizability of our findings and confines the feasibility and safety profile of 4D ICE to experienced teams. Our study did not quantitively measure the procedural duration and radiation exposure, future prospective studies including both ICE-guided and non-ICE procedures are needed to evaluate differences in procedural duration and radiation exposure. Furthermore, all procedures were performed via a femoral venous approach, so our data does not address the feasibility or safety of ICE via alternative access routes. Additionally, our data addresses acute-only outcomes and does not reflect long term implications or outcomes. Nevertheless, given the successful use of ICE at other institutions, we believe our findings are likely to be reproducible in similarly experienced centers.

## CRediT authorship contribution statement

**Eihab Ghantous:** Writing – review & editing, Writing – original draft, Visualization, Validation, Supervision, Software, Resources, Methodology, Formal analysis, Data curation, Conceptualization. **Hans Gao:** Validation, Data curation. **Carlos Sisniega:** Validation, Data curation. **Angela Li:** Writing – review & editing, Validation, Conceptualization. **Jamil Aboulhosn:** Writing – review & editing, Writing – original draft, Validation, Data curation, Conceptualization.

## Disclaimer

Dr. Aboulhosn serves as Deputy Editor in the IJC-CHD journal but had no involvement in the handling of this paper.

## Funding

none.

## Declaration of competing interest

The authors declare the following financial interests/personal relationships which may be considered as potential competing interests: Jamil Aboulhosn reports a relationship with Edwards Lifesciences Corporation that includes: consulting or advisory and funding grants. Jamil Aboulhosn reports a relationship with Medtronic Inc that includes: consulting or advisory and funding grants. Jamil Aboulhosn reports a relationship with Venus Medtech (Hangzhou) Inc. That includes: funding grants. Jamil Aboulhosn reports a relationship with Siemens that includes: consulting or advisory. If there are other authors, they declare that they have no known competing financial interests or personal relationships that could have appeared to influence the work reported in this paper.
